# Evaluation design of a systematic, selective, internet-based, *Chlamydia *screening implementation in the Netherlands, 2008-2010: implications of first results for the analysis

**DOI:** 10.1186/1471-2334-10-89

**Published:** 2010-04-07

**Authors:** Ingrid VF van den Broek, Christian JPA Hoebe, Jan EAM van Bergen, Elfi EHG Brouwers, Eva M de Feijter, Johannes SA Fennema, Hannelore M Götz, Rik H Koekenbier, Sander M van Ravesteijn, Eline LM Op de Coul

**Affiliations:** 1 Epidemiology & Surveillance Unit, Centre for Infectious Disease Control, National Institute of Public Health and the Environment, Bilthoven, the Netherlands; 2 Department of Infectious Diseases, South Limburg Public Health Service, Geleen, The Netherlands; 3 STI AIDS Netherlands, Amsterdam, the Netherlands; 4 Cluster of Infectious Diseases, Department of Research, Online Research and Prevention Unit, Amsterdam Health Service, Amsterdam, the Netherlands; 5 Division of Infectious Disease Control, Rotterdam Rijnmond Public Health Service, Rotterdam, the Netherlands

## Abstract

**Background:**

A selective, systematic, Internet-based, *Chlamydia *Screening Implementation for 16 to 29-year-old residents started in three regions in the Netherlands in April 2008: in the cities of Amsterdam and Rotterdam and a more rural region, South Limburg. This paper describes the evaluation design and discusses the implications of the findings from the first screening round for the analysis. The evaluation aims to determine the effects of screening on the population prevalence of *Chlamydia trachomatis *after multiple screening rounds.

**Methods:**

A phased implementation or 'stepped wedge design' was applied by grouping neighbourhoods (hereafter: clusters) into three random, risk-stratified blocks (A, B and C) to allow for impact analyses over time and comparison of prevalences before and after one or two screening rounds. Repeated simulation of pre- and postscreening *Chlamydia *prevalences was used to predict the minimum detectable decline in prevalence. Real participation and positivity rates per region, block, and risk stratum (high, medium, and low community risk) from the 1st year of screening were used to substantiate predictions.

**Results:**

The results of the 1st year show an overall participation rate of 16% of 261,025 invitees and a positivity rate of 4.2%, with significant differences between regions and blocks. Prediction by simulation methods adjusted with the first-round results indicate that the effect of screening (minimal detectable difference in prevalence) may reach significance levels only if at least a 15% decrease in the *Chlamydia *positivity rate in the cities and a 25% decrease in the rural region after screening can be reached, and pre- and postscreening differences between blocks need to be larger.

**Conclusions:**

With the current participation rates, the minimal detectable decline of *Chlamydia *prevalence may reach our defined significance levels at the regional level after the second screening round, but will probably not be significant between blocks of the stepped wedge design. Evaluation will also include other aspects and prediction models to obtain rational advice about future *Chlamydia *screening in the Netherlands.

## Background

*Chlamydia trachomatis *is a sexually transmitted bacterial infection that remains asymptomatic in most cases, but it can cause serious complications later in life, especially for women. *Chlamydia *screening programmes have been introduced to improve case finding, but screening apparently healthy people remains an area of considerable debate among public health researchers, sexually transmitted infection (STI) specialists, and policy makers [1-3]. Objectives of a screening programme involve not only the level of individual health (reducing complications through early diagnosis and treatment), but also the level of public health (reducing transmission within the population). The body of evidence for the effectiveness of population screening programmes is limited [4,5], and no convincing evidence of the effectiveness of screening young people for opportunistic *Chlamydia *exists [3,6]. Randomized controlled trials (RCTs) that specifically focus on population impact and thorough evaluations might provide the evidence needed to justify widespread population screening.

### *Chlamydia *screening in the Netherlands

During 2002 and 2003 a pilot *Chlamydia *screening program was undertaken in the Netherlands (Pilot *Ct*) which showed a relatively high *Chlamydia *prevalence, especially in highly urbanized regions [7]. These results led the Dutch Ministry of Health to consider a national *Chlamydia *screening programme on condition that sufficient insight into regional differences in prevalences and proven effectiveness, cost-effectiveness, and feasibility of screening would be obtained [8,9]. To ascertain this, a *Chlamydia *screening programme for 16 to 29-year-old residents started in Amsterdam, Rotterdam, and South Limburg in April 2008. This is the first large-scale intervention that pilots a selective, systematic, Internet-based, *Chlamydia *screening, and it provides a unique opportunity to gather evidence of the effectiveness of screening. The set-up of the current screening programme facilitates the in-depth evaluation necessary to decide whether and how a national roll-out of *Chlamydia *screening in the Netherlands can take place in the future. The use of a randomized stepped wedge approach - as an alternative to an RCT - allows for the study of the impact of a screening programme with at least two screening rounds on outcome parameters such as the population prevalence of *Chlamydia *and self-reported pelvic inflammatory disease (PID). Here, we clarify the design of our evaluation and discuss the implications of the results of the first round for the final evaluation after completion of two screening rounds.

## Methods

### Overview of the screening programme

The *Chlamydia *Screening Implementation (CSI) is being implemented by the Public Health Services in Amsterdam, Rotterdam, and South Limburg. STI AIDS Netherlands is coordinating the programme. In collaboration with these implementing parties, the Centre for Infectious Disease Control at the National Institute for Public Health and the Environment (RIVM) will provide process and impact evaluations. The background and set-up of CSI (including a flowchart) are explained in detail elsewhere [10,11]. Briefly, the features are:

▪ Design: selective, systematic, population-based screening.

▪ Invitees: in the 1st year, more than 261,000 people aged 16 to 29 years, obtained from the population register, are invited to participate. In Amsterdam and Rotterdam where the population is dense, all sexually active people are encouraged to participate, but in South Limburg where the population is less dense, eligibility for screening depends on the individual's score on a questionnaire (including e.g. sexual history, residence area, ethnic background, and symptoms) related to the expected *Chlamydia *risk [12].

▪ Setting: invitation letters containing the website address http://www.chlamydiatest.nl and a personal login code are sent by mail. Communication and screening procedures are Internet based. Home sampling kits (urine or vaginal swab) can be requested through this website.

▪ Intervention: *Chlamydia *test; advice and referral letter for treatment (for *Chlamydia*-positive participants and current partners). Opportunity to notify former partners anonymously through the website http://www.chlamydiatest.nl.

▪ Follow-up: repeated invitation in two consecutive periods of 1 year. Chlamydia-positive participants automatically receive a test package 6 months after the first test.

▪ Laboratory procedures: nucleic acid amplification techniques (NAAT) in three regional accredited laboratories [10].

▪ Data collection: data are stored in a central database. For each invitee, demographic information from the municipal database is combined with automatically generated data from screening 'steps'. The data are uploaded from the laboratories and on-line questionnaires filled in by the participants. The data include age, gender, and client characteristics such as self-reported sexual history and clinical symptoms.

### Evaluation

The evaluation of the CSI consists of two main components, both including various substudies:

▪ A process evaluation, which examines the extent to which the programme is operating as intended by assessing programme operations and determining how well the target population has been reached (non response and acceptability). The design of the process evaluation and first results are described elsewhere [11].

▪ Impact evaluation, which aims to assess the effectiveness of screening on the prevalence of *Chlamydia *in the target population. We describe this part of the evaluation in more detail in this paper. The cost-effectiveness will be addressed in another paper.

### Impact evaluation

#### Participation and positivity rates evaluated in the stepped wedge design

**Figure 1:**
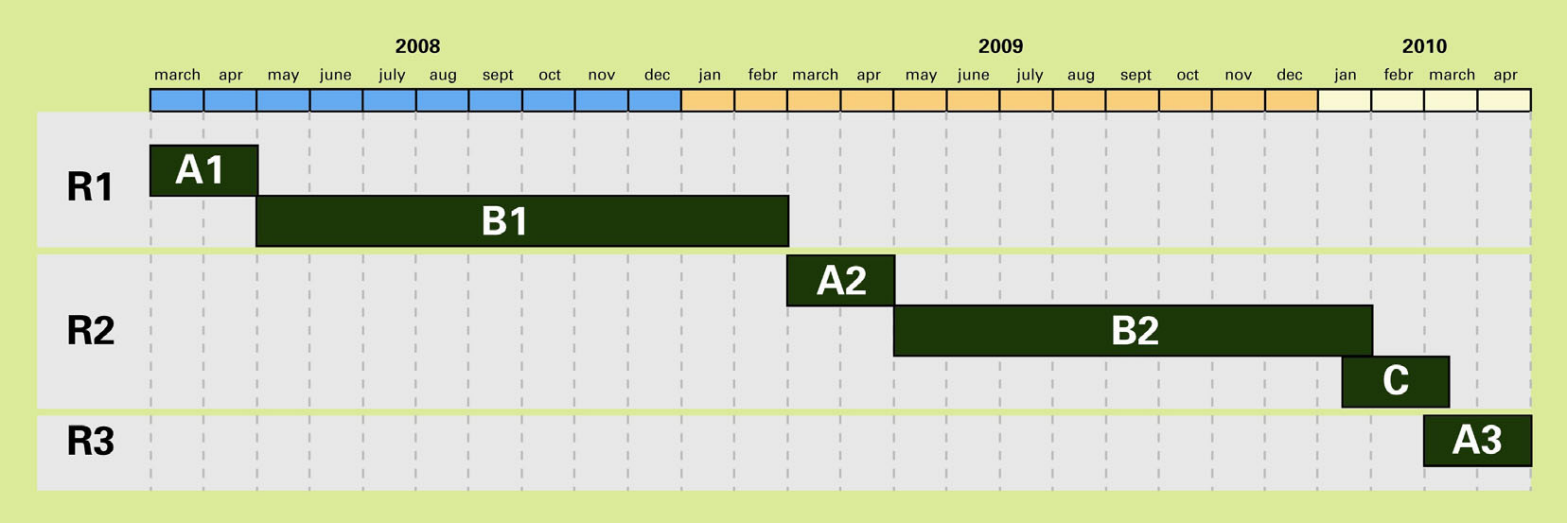
**Stepped wedge design: schematic of the phased implementation of *Chlamydia *Screening Implementation in three blocks (A,B, and C)**. The design enables comparison of prescreening (A1 and B1) and post-screening prevalences after the first round (A2 and B2) and after the second round (A3)

To evaluate the effects of screening on the main outcome parameters, i.e. the participation rate (the proportion of invitees who send a sample to the laboratory and the estimated prevalence of *Chlamydia*), we chose a phased implementation of the screening for randomly selected groups. This stepped wedge design is defined as *a sequential roll-out over a number of periods; by the end of the programme, all those eligible will have received an invitation to participate, even though the order is determined at random *[13]. The design enables estimating (a) the effect of one or two screening rounds on the estimated population prevalence of *Chlamydia*, (b) time trends in the participation rates of the screening rounds, and (c) indirect effects of the screening on groups not targeted in the first round (spill-over effect). It will make estimating the pre- and postscreening *Chlamydia *prevalences in these regions possible. The *Chlamydia *positivity rate and the self-reported PID in the screened population will be assessed within the stepped wedge roll-out of the screening in three randomly selected subgroups of the target populations of each region (Figure 1). In both Amsterdam and Rotterdam, one smaller group (block A, one-sixth of the target population) will be offered screening three times. The largest group (block B, two-thirds of the population) will be offered screening twice, and another smaller 'control' group (block C, one-sixth of the population), only once, in a later phase. Due to smaller populations to invite in South Limburg, the three blocks are equal in size to maximize the power of comparisons between groups. We prefer the step-by-step implementation for the purpose of logistics, since it is difficult to cover all regions at once. The speed of the roll-out of the intervention is adjusted to the number of invitations that can be handled per day and the laboratory capacity.

To compare pre- and postintervention groups after one and two screening rounds, the *Chlamydia *positivity rates from blocks A1 and B1 of the first round (R1) will be used to estimate the *Chlamydia *prevalences that will serve as proxies for the prevalences in the target population before the screening started (Figure 1). These 'prescreening prevalences' from A1 and B1 can be compared with the 'postscreening prevalences' after the first round (A2 and B2) and after two rounds (A3). The prevalence estimate from the control group (C) is considered a proxy for the *Chlamydia *prevalence in a group that has not received the intervention and will also be used to estimate 'secular time trends' in nonintervention areas; i.e. the effect of screening on the *Chlamydia *prevalence in an unscreened population.

#### Cluster randomization

Each region consists of different neighbourhoods (hereafter named clusters) which we used as the level of randomisation. These clusters were defined by the 'community risk level' as high, medium or low on the basis of age-profile (proportion of 16-29 years old), ethnic profile (proportions of Surinamese and Antillean residents (known risk groups) [14], and income profile (proportion in lower income category). In South Limburg, the level of urbanization was also taken into account.

The order of invitations is thus cluster randomized instead of individually randomized. We expect the effect of detection and treatment of *Chlamydia *cases to be higher when geographical clusters are submitted to screening in a short period. We predict cluster randomization to cover more social networks and sexual partnerships compared to individual randomization. Furthermore, inviting all young people in one cluster at the same time will minimize stigmatization and prevent questions such as 'why me?'.

**Figure 2:**
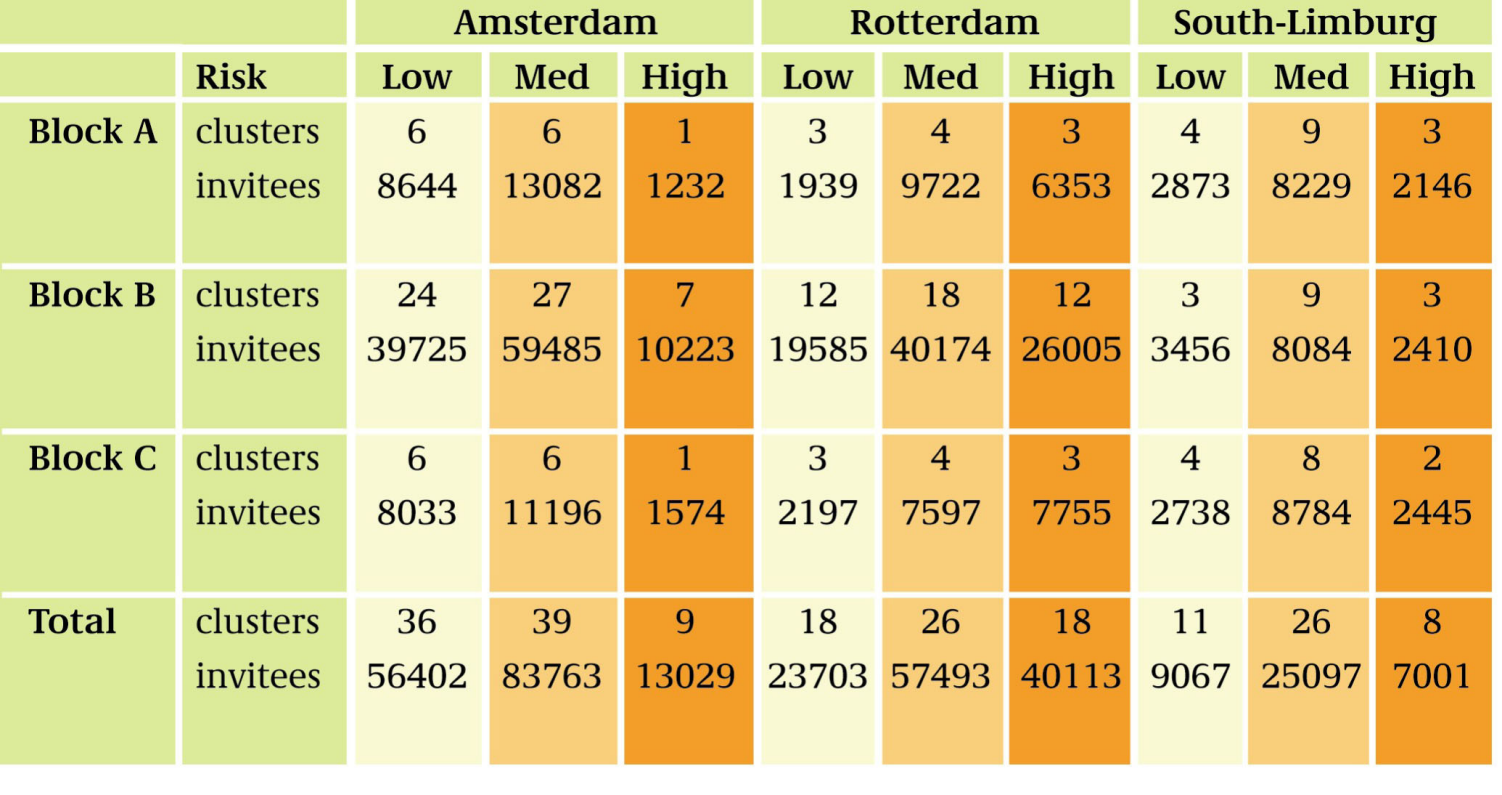
**Number of clusters and invitees, by region, block and community risk level**.

**Figure 3:**
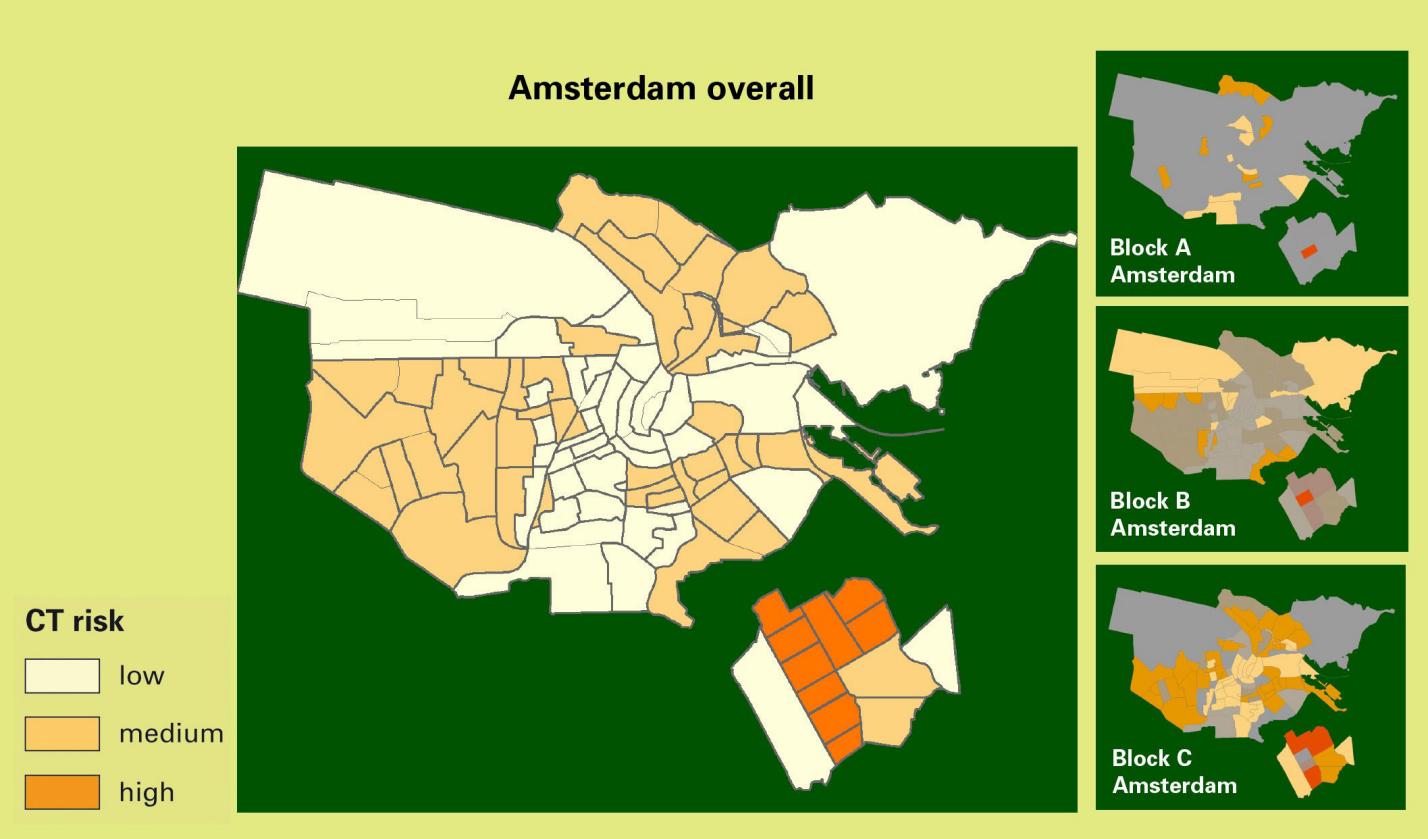
**Risk stratification and cluster randomization: map of Amsterdam**. The map shows the division into clusters (neighbourhoods) with an indication of *Chlamydia *risk levels and randomized stratification into blocks A, B, and C.

**Figure 4:**
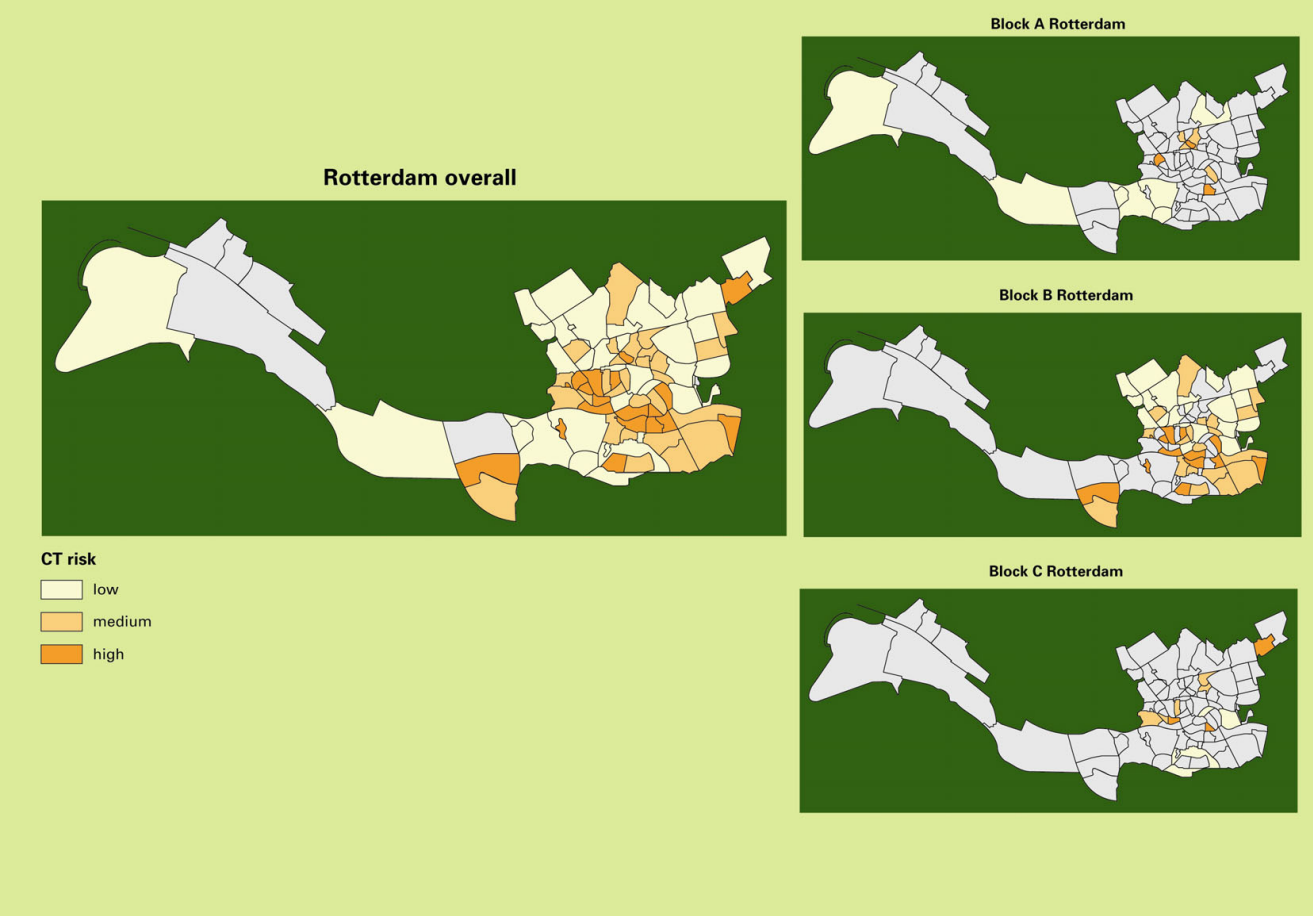
**Risk stratification and cluster randomization: map of Rotterdam**. The map shows the division into clusters (neighbourhoods) with an indication of *Chlamydia *risk levels and randomized stratification into blocks A, B, and C.

Randomization of clusters is stratified within the blocks A, B, and C by the expected risk levels for *Chlamydia*, so that the proportion of high-, medium-, and low-risk clusters was similar for the blocks in each region (Figure 2). Figure 3 and 4 illustrate this with the stratification of clusters in Amsterdam and Rotterdam.

#### Power estimates and simulations of outcome

A significant decline in the *Chlamydia *prevalence after one or two screening rounds would establish the effectiveness of the screening programme. The magnitude of the decline that can be assessed will depend on the degree of participation and the *Chlamydia *positivity rates. We tested the expected power of our design for comparisons of prevalences with Monte Carlo simulations of the outcomes of the screening rounds 1 and 2 for each of the regions (Amsterdam, Rotterdam, and South Limburg) and for the blocks A, B, and C. The purpose of these simulations was to determine how many people have to participate in the screening for a certain decline in *Chlamydia *prevalence to be detectable.

Based on the Pilot *Ct *[7], we anticipated an overall participation rate of 30% and an average positivity rate of 4-6%. For the power calculations, the following assumptions were made: (a) in all clusters, a similar fraction of the population participates in the screening; (b) the participating population is a random selection of the invited population; (c) the hypothetical prevalence rates per cluster vary from 0 to 10%, normally distributed around the average; and (d) after screening, we expect a decline in *Chlamydia *prevalence.

Each simulation included the following steps: (1) simulation of cluster prevalence between 0 and 10% with an average of 4%, 5%, or 6% before screening; (2) simulation of cluster prevalence with an average below 4%, 5%, or 6% after screening; and (3) calculation of the significance of the differences in prevalences. This procedure was repeated 5000 times for various sample fractions (participation rates). The percentage of simulations with significant differences (power) was calculated. Finally, contour plots were made with expected participation rates against the decline in mean prevalence after one or two screening rounds, per region, (a) for the whole target population in each region and (b) for the subpopulations of the three blocks in the stepped wedge design (see results).

#### Comparison of participants and non participants

Profiles of participants and non participants will be studied to evaluate how people perceive the screening programme and whether high-risk groups have been reached. During the screening, detailed background information about participants and non participants is collected by means of online questionnaires and mail. Screening test results are monitored in a central database, which includes the data from the online questionnaires. Participants who log in to the website are asked about education, sexual experience, and history of STI. After the test results have become available, participants who tested positive are asked about their doctor's consultation and *Chlamydia *treatment for themselves and their current partners; they are also asked whether they informed recent ex-partners and whether they made use of the option to enlist them for a screening invitation.

A sample of participants (*n *= 5500) are to receive an additional e-mail questionnaire in which they can give their opinion of the set-up of the screening and the information provided so that we can assess the acceptability of the screening procedures. In addition, a sample of non-participants (*n *= 13,500) are to be approached by surface mail to answer a questionnaire about why they did not participate, their opinion of the screening, and personal characteristics.

#### From Chlamydia positivity to Chlamydia prevalence

We will estimate the *Chlamydia trachomatis *prevalence in the target population from the positivity rates by extrapolation of the screening participants' results to the whole target population, by comparing the participant and non participant characteristics including age, gender, region of origin, and sexual behavioural information. Other factors such as re-infection rates and treatment will also be noted.

In South Limburg, we will use an extra selection tool aimed at including participants with a potentially higher risk of *Chlamydia *to increase the cost-effectiveness of screening in areas with a lower population density and a lower prevalence of *Chlamydia*. This tool will be evaluated for its effect on the participation and positivity rates of subgroups in the population [12,13].

Furthermore, other data sources in the intervention regions will be more widely studied for *Chlamydia *prevalence and sexual behaviour in the Netherlands. These data sources include the STI surveillance in STI centres, general practitioner (GP) networks, and laboratory and hospital registers. Combined effects from screening outcomes and *Chlamydia-*testing at these sites will be taken into account. Similar analyses will be conducted for self-reported PID. Trends of PID will also be studied within the surveillance networks of GPs and hospitals.

#### Prediction model for future effects of screening on prevalence

Data collected during the screening will be used in combination with other datasets and literature data (e.g. on sexual behaviour, transmission risks, and the likelihood of developing complications such as PID) for an epidemiological model of transmission dynamics of *Chlamydia *[15,16]. This dynamic epidemiologic model will be used to compute the estimated numbers of new infections and avoided complications for different intervention options. The model - a simulation model based on individuals - uses data such as age, gender, sexual activity (high or low), status of infection (not infected, symptomatic, or asymptomatic), duration of infection, the number and identity of partners (casual or steady), and the duration of relationships. The annual incidences, as calculated with the model, are then used as input for a Markov model that describes the potential long-term consequences of infection [17-19]. The variation of input parameters will help us address important questions about who to screen and how frequently to do so.

The screening programme and evaluation have been approved by a Medical Ethics Committee of the VUmc in Amsterdam (METc number: 2007/239). Participants provide online informed consent.

## Results

During the first screening round, from April 2008 to February 2009, a total of 261,025 people were invited (blocks A and B). Altogether, 52,741 people (20.2%) requested a *Chlamydia *test package online, and 41,638 people (16.0%) participated in the screening by sending a sample to the laboratory.

### Comparison of findings per region and per block of the stepped wedge design

**Figure 5:**
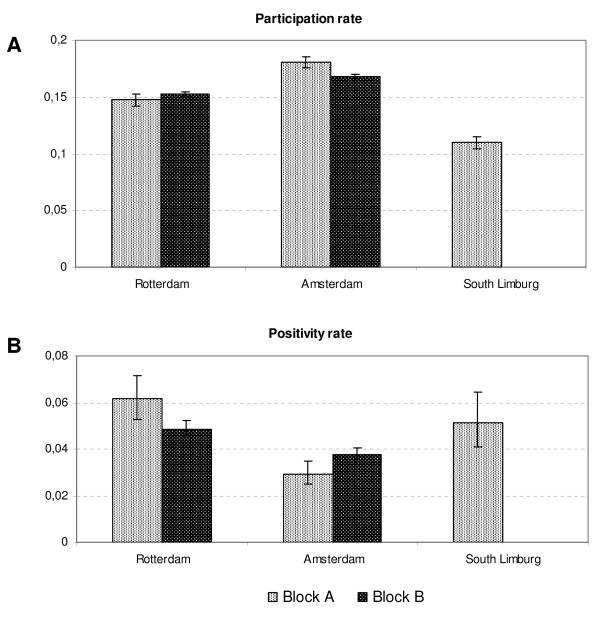
**Participation rates (A) and positivity rates (B) in year 1 of the *Chlamydia *screening, per region and for blocks A and B of the stepped wedge design**. Error bars show 95% confidence intervals.

The participation rates were 17.0% in Amsterdam, 15.2% in Rotterdam, and 11.0% in South Limburg - significantly different rates in the three regions (χ^2^: *p *< 0.001; Figure 5 and Table 1). The participation rate in South Limburg was lower because of the selection-by-risk score: 22.5% of the South Limburg invitees went online to fill in the risk score, of whom 63% had a score high enough to request a test package. The positivity rate among participants in Amsterdam (3.6%) was lower than in Rotterdam (5.1%). In South Limburg, the positivity rate was also 5.1% due to the selective inclusion of people at risk. Block A and block B in Amsterdam had significantly different participation and positivity rates (χ^2^: *p *< 0.001). In Rotterdam, the participation rates in the two blocks were similar, but the positivity rates differed (*p *= 0.01).

**Table 1:** Participation and positivity rates in the first screening round and minimal detectable difference in positivity between rounds 1 and 2 at a power of 80%.

		Number invited	Participation rate % (95% CI)	Positivity rate % (95% CI)	Detectable decline of positivity rate (in % after screening)	
**Amsterdam**	**Block A**	25,121	18.0 (17.5-18.5)	2.9 (2.5-3.5)	Block A versus block B	0.9
	**Block B**	114,937	16.8 (16.6-17.0)	3.7 (3.5-4.0)	Block A versus block C	1.2
	**Overall**	140,058	17.0 (16.8-17.2)	3.4 (3.8-0.2)	Round 1 to round 2	0.5

**Rotterdam**	**Block A**	17,929	14.8 (14.2- 15.3)	6.2 (5.3-7.2)	Block A versus block B	1.4
	**Block B**	89,877	15.3 (15.0-15.5)	4.9 (4.5- 5.2)	Block A versus block C	1.8
	**Overall**	107,806	15.2 (15.0-15.4)	5.1 (4.7-5.4)	Round 1 to round 2	0.8

South Limburg*	**Block A**	13,124	11.0 (10.5-11.5)	5.1 (4.1-6.4)	Block A versus block B	1.9
	**Overall **(only block A in first round)				Round 1 to round 2	1.2

* with application of a selection based on risk scores

### Findings per cluster and community risk level

**Figure 6:**
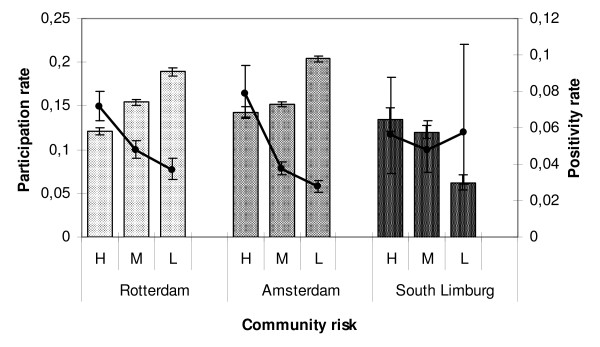
**Participation rates (bars) and positivity rates (lines) in year 1 of the *Chlamydia *screening per region and for the three community risk levels estimated on the basis of demographic characteristics**. Error bars show 95% confidence intervals; H = high, M = medium, L = low.

The participation and positivity varied greatly per geographical cluster. In Amsterdam, participation rates varied from 8% to 26% and positivity rates from 1% to 15% per cluster. In Rotterdam, the ranges were 8% to 25% and 1% to10%, respectively, and in South Limburg 5% to 19% and 2% to 9%, respectively. The community risk estimates per cluster, based on demographic characteristics of age, ethnicity, level of income, and urbanization, resulted in three distinct groups of clusters in each region. High-risk clusters in Rotterdam and Amsterdam had lower participation rates, but higher positivity rates than medium- and low-risk clusters (*p *< 0.001). In South Limburg, where participants were selected by risk score, the high- and medium-risk clusters had higher participation rates than the low-risk clusters (*p *< 0.001), while the positivity rates did not differ (*p *= 0.8; Figure 6).

### Prediction of detectable decrease after screening

**Figure 7:**
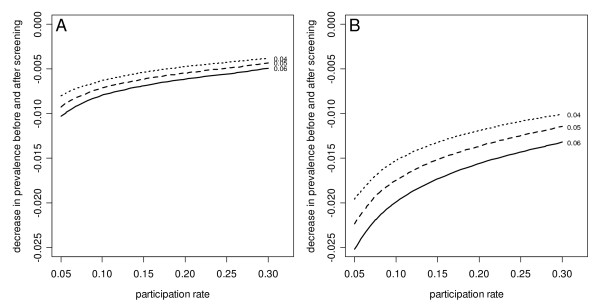
**Detectable differences in prevalences between screening rounds 1 and 2**.  Power levels are noted on lines, and 4%, 5%, and 6% prevalence rates in screening round 1 (*p*_0_) are presented as a function of the participation rate (sample fraction): **A, **simulations for Amsterdam, overall and **B, **comparison of blocks A and C.

At forehand, contour plots were made with expected participation rates (30%) against the decline in mean prevalence after one or two screening rounds, for each region and for the subpopulations of the blocks in the stepped wedge design (Figure 7, example Amsterdam). On the assumption of an overall *Chlamydia *prevalence of 5% at the start of the screening, these plots showed that the cluster set-up has sufficient power (80%) to detect a reduction of the *Chlamydia *prevalence from 5% in screening round 1 to 4% in screening round 2, provided that the participation rates were at least 10% in Amsterdam and Rotterdam and at least 25% in South Limburg. For sufficient power to detect a 1% difference in prevalence between the three blocks, the participation rates should be higher: 25% to 45%. The effect of screening on the prevalence rate should be robust if participation rates of the overall regions are between 10% and 20%.

By using the real participation- and *Chlamydia *positivity rates from the first screening round (Table 1), we determined the minimal decline needed to find a significant effect of screening on *Chlamydia *positivity. In Amsterdam, a decrease of overall positivity from 3.4% in the first round to 2.9% in the second round (a proportional decline of 15% from the first round positivity) would be significant; in Rotterdam a decline from 5.1% to 4.3% (16%) and in South Limburg from 5.1% to 3.9% (24%) would be significant. However, to show differences between the blocks, a steeper decline is needed, i.e. for comparing block A with block C in Amsterdam, a decrease of 1.2% in positivity (35% of that in the first round) is needed in the second round at a participation rate of 17%. In Rotterdam and South Limburg, similar proportional declines are necessary at current participation rates to prove any effect of one screening round comparing the positivity in blocks A, B, and C.

### Response to questionnaires

The response rate to the general questionnaire (at login on the website) was high: 60% of the participants. In total, 30,517 people responded, and most of them (82%) were participants, but some requested a test package and did not send it back (16%), and 2% filled in the questionnaire without requesting a package. About 750 people who tested positive (43%) completed the questionnaire about treatment. The acceptability questionnaire had a response of 63% (*n *= 3500), and the nonresponse questionnaire, 15% (*n *= 2050). The women's response rate was higher than that of men for all questionnaires. Younger people (< 20 years old) more often replied than did older people (20-29 years old).

## Discussion and Conclusions

Invitations for the first round of the *Chlamydia *screening in Amsterdam, Rotterdam, and South Limburg were completed by the end of February 2009. Nearly one of six 16 to 29-year-olds in the three participating regions have been screened in this first round, and one in 25 participants tested positive for *Chlamydia*. The final impact evaluation of the programme is expected when two screening rounds have been completed in 2010. However, the findings from the 1st year allow us to review (the expected power of) our evaluation design.

The overall participation (16%) was lower than the 30% in the Pilot Ct (both register based) which was expected as our programme is selective in its nature: only for sexually actives and for Limburg a further selection by the prediction rule was incorporated in the programme. Moreover, the programme used test kit requests via internet instead of directly sending test kits. We want to caution about (inter)national comparisons of participation rate as programme designs may differ and denominators and test kit offering may not be comparable [10]. For instance, in opportunistic programmes, acceptance of test-offer may be presented as coverage; sometimes denominators include only sexually actives or are not population-based but reflect attendees of health facilities. The overall 16% participation rate is based on the total number of invitees which includes people who are not sexually active yet.

The overall positivity rate of 4.2% was higher than that of the pilot CT study (2.0% overall and 3.2% in highly urbanized regions) [7], the Amsterdam programme (3.4%) [20] and the UK programme (3.1%) [21], suggesting an adequate (self) selection which will benefit cost-effectiveness of the programme.

Using simulations, we have confirmed that, with participation and positivity rates as observed in the 1st year of screening, we will be able to show a significant 'overall' effect of one screening round on the *Chlamydia *prevalence if it decreases by about 15% in the cities and 25% in South Limburg. At the level of the blocks in the stepped wedge design, an effect might not be detectable with statistical significance after two screening rounds, unless the prevalence diminishes by one-third or more.

The effect of screening might better be evaluated separately in the three regions of Amsterdam, Rotterdam, and South Limburg because participation and positivity rates in the regions were significantly different. Blocks A and B of the cluster-randomized stepped wedge design also showed different participation and positivity rates in this first round. The randomization of clusters was stratified by risk level, but this apparently did not result in completely similar groups within one region. These differences should be taken into account, and multilevel analysis should be used to compare pre- and post-screening prevalence (extrapolation of findings in blocks A, B, and C).

There was a large variation in participation and positivity rates at the lowest level among the clusters. Beforehand, we assumed a positivity rate per cluster between 0% and 10% (for simulations). Most clusters remained within this range (only one cluster in Amsterdam had a positivity rate above 10%). The assignment of the risk levels (high, medium and low community risk, based on the criteria of age, ethnicity, income profile, and urbanization) created different groups of clusters, at least in the two cities. Participation was lower and positivity was higher in high-risk clusters, as can be expected, and as was also found in the UK [21]. Studying the effect of screening within these different risk levels will provide food for thought for further population screening targets.

### Limitations

The stepped wedge design facilitates the analysis of the effect of multiple screening rounds on test positivity in the light of modelling *Chlamydia *transmission and estimating cost-effectiveness, but will also have limitations. First, equal sizes of the three blocks in Amsterdam and Rotterdam would have increased the power of the comparisons between blocks, but would deviate too much from the agreed two screening rounds for everyone. Secondly, potential bias is caused by the fact that the participation and the receiving of a *Chlamydia *positive or -negative test result in the first screening round may affect the participation in the subsequent rounds. This can be reviewed by comparing consistent non responders, repeat participants, and one-time participants.

The characteristics of participants and non responders (gender, age, ethnic background, sexual activity, et cetera) will be used to predict the *Chlamydia *population prevalence from the *Chlamydia *positivity rate by modelling. The success of these predictions will depend on the outcomes of the non response study: non responders are the largest group in our population, and estimating the proportion of those not responding because they are not at risk (because they are not, or not yet, sexually active or are in a long-term faithful relationship) is very important. One in 15 non responders received a questionnaire, and 15% replied. For those, information about the sexual history is available. However, basic characteristics (gender, age, ethnic group, and cluster) from the population registers are available for all non responders. The participant response rates to the online general questionnaire, the acceptability study via e-mail (60% or more) and the online treatment questionnaire were higher than response rates to the treatment questionnaire among those who tested positive (43%). The questionnaires will provide detailed insight into 'who participated when and why' (and who did not), as well as information about behavioural characteristics such as sexual behaviour, number of sexual partners, and condom use.

### Opportunities

The stepped wedge design has the advantage that the first screened block(s) can be used for a baseline prevalence measurement, so that extra surveys (before and after screening) that could seriously interfere with participation and positivity in the screening population, could be avoided.

A reduction in *Chlamydia *transmission attributable to screening would provide good primary evidence of effectiveness [3]. Further interpretation and modelling are needed to predict the effects of multiple (more than two) screening rounds. Existing predictive models [15,16] will be updated with the findings from the screening and recent literature. Much information will be obtained from the CSI database, which will include around 300,000 invitees and, as expected on the basis of the first results, 40,000 to 50,000 participants per year. The large-scale CSI-programme, as implemented in the Netherlands, provides a unique opportunity to assess the effectiveness of a systematic, population-based screening offered to young people for reducing the *Chlamydia *prevalence - and self-reported PID prevalence - in the screened population and the population at large.

The outcomes can be compared to other ongoing initiatives such as opportunistic screening as practised in the UK, Sweden, and the USA [22], population-based studies such as those in Sweden [23], Denmark [5], and the Netherlands [7,24], and various RCTs [4,5]. This may provide the necessary evidence for the effectiveness of register-based programmes [3]. In conclusion, we will use a combination of methodologies to investigate innovative aspects of the design of CSI. We expect the project to yield new insights into the impact of *Chlamydia *screening, epidemiological trends, and screening that makes use of the Internet. The comprehensive evaluation will enable national health authorities to decide whether to implement nationwide *Chlamydia *screening in the Netherlands.

## Competing interests

The authors declare that they have no competing interests.

## Authors' contributions

All authors are part of the CSI Project Group*, which is the group involved in the conception and design of the *Chlamydia *Screening Implementation. IB and EC developed the methodology of evaluation, analysed and interpreted the data, and drafted the manuscript. Other authors were involved in the roll-out of the programme (including data acquisition) and contributed to drafting and revision of the paper. JB and EF were responsible for the coordination of the screening in the three regions; CH and EB, for the screening in South Limburg, JF and RK in Amsterdam, and HG and SR in Rotterdam. All authors read and approved the final manuscript.

* Current composition of the CSI project group: JEAMB, IVFvdB, EEHGB, JSAF, HMG, CJPAH, RHK, ELM OdC, LLP, SMvR.

## Pre-publication history

The pre-publication history for this paper can be accessed here:

http://www.biomedcentral.com/1471-2334/10/89/prepub
